# Diabetes Management after a Therapeutic Education Program: A Qualitative Study

**DOI:** 10.3390/healthcare10081375

**Published:** 2022-07-24

**Authors:** Rocío Romero-Castillo, Manuel Pabón-Carrasco, Nerea Jiménez-Picón, José Antonio Ponce-Blandón

**Affiliations:** Centro Universitario de Enfermería de Cruz Roja, Universidad de Sevilla, Avenida de la Cruz Roja, nº 1, 41009 Seville, Spain; rocio.romero@cruzroja.es (R.R.-C.); mpabon@cruzroja.es (M.P.-C.); japonce@cruzroja.es (J.A.P.-B.)

**Keywords:** type 1 diabetes mellitus, qualitative study, health promotion, self-care, nursing

## Abstract

(1) Background: Structured education has been used in patients treated with insulin, promoting their ability to adhere to and self-manage their treatment. We aimed to know the perception and adherence to the recommendations on the management of diabetes in type 1 diabetes patients after participating in a therapeutic education program. (2) Methods: A descriptive qualitative study followed the Standards for Reporting Qualitative Research guidelines. Patients with type 1 diabetes participating in a therapeutic education program were recruited from February to April 2022. Semi-structured, in-depth interviews were used, and transcriptions were analyzed using the inductive qualitative content analysis method. The final sample consisted of 18 type 1 diabetes mellitus patients. (3) Results: A number of patients with type 1 diabetes said that they had improved their glycemic control after participating in the therapeutic education program. Some patients recognized that the chronic disease and the complications complexity generated stress and anxiety. This influenced their usual life, at work, and their interpersonal relationships. (4) Conclusions: In terms of satisfaction, the majority of patients reported a good perception of the quality of the organization, the professionals involved, and the contents of the program. The physical and psychosocial benefits of self-care training have been demonstrated.

## 1. Introduction

Diabetes mellitus (DM) is one of the most complex chronic pathologies worldwide in the 21st century [1]. Managing diabetes can lead to a psychological burden for patients with DM and their families. Diabetes distress has been found to be the strongest independent predictor of metabolic control among patients with type 1 diabetes [2]. Diabetes distress is related to emotional burden, anxiety, worries, and stressors that arise from living with a complex condition such as diabetes [3].

Noncommunicable diseases are chronic diseases of long duration and generally slow progression. In the community population, the disease control of most noncommunicable diseases patients (hypertension, diabetes mellitus, and coronary heart disease) relies on self-management, but the disease control rate does not meet the expectations of health systems. Health education generally contains education on rational drug usage, regular consultation, psychological support, and lifestyle guidance [4].

In recent years, there has been a growing recognition that therapeutic education is an important part of the integrated treatment care for diabetes mellitus. Structured education has been used in patients treated with insulin, promoting their ability to adhere to and self-manage their treatment [5,6]. The multiple insulin injections, the calculation of carbohydrates, exercise and hypoglycemia, and hyperglycemia prevention and treatment require a high level of knowledge and commitment from patients.

One of the main elements of a successful therapeutic education is the commitment and alliance that is established between the professional and the patient. Many of the patients struggle after the course to implement and maintain the skills learned and achieve optimal blood glucose levels, in some cases being far from the objectives [7]. Diabetic patients participating in structured educational programs obtain improvement in blood glucose levels, less hypoglycemia, fewer emergency call-outs and hospital admissions, and improved quality of life, but many find it hard to sustain self-management [8]. One of the National Institute of Health and Care Excellence (NICE) recommendations says that “programmes should promote active learning and be adapted to meet the needs, choices and learning styles of people with diabetes and integrated into routine diabetes care” [9].

Numerous studies have proven the benefits of Diabetes Self-Management Education and Support (DSMES), which include improved clinical outcomes and quality of life while reducing hospitalizations and health care costs [10,11]. To demonstrate the benefits of DSMES, it is important to measure relevant clinical outcomes, patient-reported outcomes, and psychosocial and behavioral outcomes [12].

A number of factors have been associated with the success of programs: (a) Better results were obtained from more intensive programs and when there is a shorter delay between the end of the program and the measurement of results [13]. (b) Teams led by nurses achieve significant reductions in mortality [10]. (c) Better results are obtained in patient-centered care programs, with interventions based on theoretical models and with an educational itinerary structured by a professional adviser [14].

A recent clinical trial has examined the results of the *DiaLife* education program. It was obtained significantly better diabetes-related knowledge (compared to the control group) directly after the intervention. *DiaLife* education program had a positive impact on the mental well-being of relatives of people with diabetes [15]. A study conducted in Canada focusing on patient education for individuals living with diabetes demonstrated that an education intervention associated with an exercise program significantly improved patients’ disease-related knowledge, exercise, food intake, self-efficacy, and health literacy. Health literacy is an important measure in studies assessing the effectiveness of educational intervention, and this intervention was able to statistically improve this outcome, which contributed to the scarce literature on this topic [16].

A clinical trial conducted in Spain measured the effectiveness of an educational intervention on adherence to a healthy diet in diabetic patients. All participants received standardized counseling about healthy eating and physical activity [17]. Professionals encouraged the “Mediterranean diet”. It is characterized by the high consumption of vegetables, monounsaturated fatty acids, and fruits; and moderate consumption of dairy products and fish and meats. The Mediterranean diet is recognized as one of the healthiest dietary patterns, and it is associated with significant improvements in glycemic control and weight loss [18]. The results of the educational intervention demonstrated moderate efficacy in adherence to the Mediterranean diet. Greater adherence to the Mediterranean diet results in an improvement in glycemic control and insulin sensitivity, which are important factors in the management of diabetes [17].

Some therapeutic education programs have been made for patients with type 1 diabetes [19]. However, adherence to the recommendations remains low–moderate [20]. Most of the studies have made quantitative analyzes with the use of scales or questionnaires with options, so we do not have the opinion and expression of the patient with open questions in which they can express the difficulties that interfere with adherence. For this reason, this study was designed with the objective of assessing the patient’s perception of quality of life and adherence after participating in a therapeutic education program offered by nurses specialized in diabetes. A qualitative design study allows us to know the opinion of the patients as well as suggestions for improvement to continue adapting the program to the needs of our patients.

## 2. Materials and Methods

### 2.1. Study Design and Settings

The specific study setting is a Diabetes Day Hospital in Spain directed by medical specializing in Endocrinology and diabetes nurse educators. In-depth qualitative interviews were conducted in person with study participants. A thematic analysis approach was used to determine commonalities of experiences of patients with type 1 diabetes mellitus after participating in a health education program. The methodology that supports this study is content analysis. Throughout this study, we followed the consolidated criteria for reporting qualitative studies (COREQ) checklist [21]. Supplementary Material File S1 shows this checklist made for this investigation.

### 2.2. Participants

From February to April 2022, the purposive sampling method was used to invite individuals to participate in the study. Patients were recruited from the diabetes education classroom after participating in an education program. Participants were selected in person in consultations or after finishing the last day of educational sessions. It was estimated that the ideal time to conduct the interviews and assess the changes in lifestyle, the acquisition of new habits, and changes in glycemic control was between one and three months after participation in the educational program. Although the proposal for the interview, the information, and the informed consent were made in person to the patient later, a reminder of the appointment for the interview was made by telephone. The inclusion criteria for study participants were as follows: (a) confirmed diagnosis of type 1 diabetes; (b) at least 18 years of age; (c) have participated in a diabetes education program in the last six months; (d) without severe impairment in mental functions that would interfere with an in-depth interview; and (e) willing to share their personal stories. Fifty patients were contacted, of whom 22 of them were recruited after securing their informed consent. Twenty-one candidates were not interested in participating in the interviews, the reasons being distance from the hospital, job incompatibility, family problems, or shyness, and the other seven patients canceled their interview due to unforeseen family and job problems. The research staff scheduled a time for one-on-one in-depth interviews. After interviewing 18 participants, a saturation point was reached, that is, the point where no new themes emerged [22].

### 2.3. Procedure

Therapeutic education was offered in four sessions of about two hours on consecutive days in groups of four patients. The group was intended to be homogenous in terms of age and number of years of evolution of their diabetes. The level of prior knowledge was intended to be similar, for which they were given a pre-test of knowledge about diabetes treatment and nutrition before assigning them to a group. The homogeneity of the groups favors the learning of all members and makes the training needs similar, avoiding inequalities and prejudice against the most educated people in the field.

Our therapeutic education was made by nurse educators who specialized in diabetes and with extensive training experience in this field of more than five years. The training was structured and based on educational intervention protocol [23]. The content of the program was divided into four sessions:

- First session. Insulin administration and blood glucose self-analysis.
- Second session. Management of hypoglycemia and hyperglycemia.
- Third session. Healthy diet adapted to the diabetic patient.
- Fourth session. Physical exercise.

### 2.4. Qualitative Data Collection

A Spanish-speaking nurse researcher, a Doctor of Health Sciences, who did not know the objectives of the study (blind researcher) and had no prior relations with the participants, conducted the in-depth interviews from March to May 2022. We believe that this would control biases to avoid conditioning the answers of the interviewees. The nurse had undergone prior training in research, specifically qualitative research at the master’s level. She had previous training in different research studies, and she did a previous specific reminder course on the use of the *Atlas.ti* tool. In addition, the researcher is an expert in psychosocial health and interpersonal skills.

The nurse introduced herself to the patient before starting the interview, created a climate of trust, and reminded the patients that the interviews were made after the diabetes education program to assess their perceptions, changes in lifestyle, perceived difficulties, and proposals for improvement. A test interview was conducted at the beginning of the study. Each interview took about 50–60 min and was audio-recorded for transcription. The interview was conducted in a classroom dedicated to diabetes education. Only the researcher conducting the interview and the patient attended. A single interview per patient was conducted on a predefined day. The recording was exclusively audio, and the transcripts were not returned to the participants.

The interview questions were agreed upon by an expert committee. The interviews were conducted in a private and quiet consultation in the afternoon to facilitate the attendance of participants. In addition, in the afternoon, there are fewer scheduled tasks in the unit than in the morning. Patients felt confident to tell us about their experience in the education program, learning, and changes in lifestyle as well as influence on their quality of life.

Participants’ demographic data (age, gender, marital status, educational level, work status, and place of residence) were obtained at the beginning of the interview. Participants were asked the following questions:

- “Do you think that the diabetes education program has been useful to improve your glycemic control?”
- “Have you implemented the recommendations offered in your daily life? Have you had any difficulty with it?”
- “Do you consider that your quality of life and your state of mind have improved after participating in the diabetes education program?”
- “Would you improve something of the course or add some aspect or activity?”
- “Is there anything else you would like to share with me or tell me?”

### 2.5. Qualitative Data Analysis

Data analysis occurred concurrently with data collection. The audio recordings were transcribed verbatim. To ensure confidentiality, the researcher pre-assigned an ID code to each subject and removed identifiable information throughout the process. All data referring to patients as well as the recorder were stored and guarded in a safe and locked place. The list relating names to number codes was destroyed at the end of the investigation. Data analysis is done only in aggregate, and no individually identifiable information from the data collected will be published. After obtaining the transcription, we randomly selected 30% to check the reliability of the transcriptions compared to the audio tapes.

**Figure 1:**
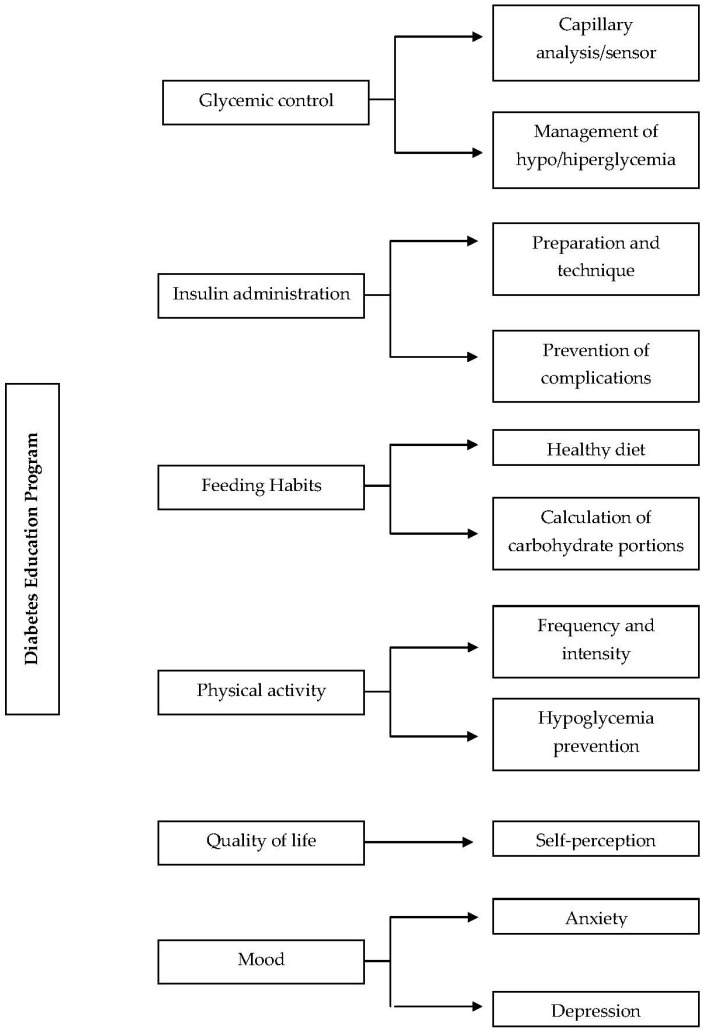
Code trees.

The software used was *Atlas.ti* (Scientific Software Development Version 7.0, 2012) to code the data and then conducted the qualitative thematic analysis. The study team then looked for concept categories and code trees related to the experiences of patients after the diabetes education course (Figure 1). Five transcriptions were randomly selected to check for coding reliability. Last, the team met to discuss and resolve discrepancies. The dependability of this study was upheld by audits conducted by external experts who were familiar with diabetes and qualitative research.

### 2.6. Ethical Considerations

The study was explained verbally to the patient, and a copy of the written informed consent was offered. All doubts were resolved, and consent could be revoked at any time. Participation was voluntary, and the data obtained were anonymized. This study obtained the permissions of those responsible for the participating entities and the approval of the corresponding ethics committee.

## 3. Results

### 3.1. Participant Characteristics

The final sample consisted of 18 type 1 diabetes mellitus patients. All patients received the same content in the therapeutic education program specialized in diabetes. The patients’ age ranged from 22 to 52 years, with an average age of 33 years; 56% were women, and 44% were men. In total, 66.7% were employed patients, 38.9% had higher education, and 61.1% had primary studies; 55.6% were single, and the rest were married. All patients were Caucasian; they resided in Spain and were Spanish speakers.

### 3.2. Thematic Results

The data obtained in this study can be organized into three themes.

#### 3.2.1. Theme 1: Usefulness of the Therapeutic Education Program in Glycemic Control

Fifteen patients with type 1 diabetes said that they had improved their glycemic control after participating in the therapeutic education program.

***Glycemic control*:** Fifteen participants learned to adjust their insulin dose based on their blood glucose level, and they calculated its sensitivity factor. They learned to resolve hypoglycemia and hyperglycemia situations.

“When I had low blood sugar, I would have a cake and they recommended me to take a liquid food with sugar, juice or soft drink. These products are absorbed faster. I have to wait fifteen minutes and I make a new control to see if the hypoglycemia has resolved. I also did not know how to adjust my dose when I had high sugar and the nurse helped me calculate my sensitivity factor.”

(42 years old, female, married)

“I sometimes skipped meals so I didn’t have to take insulin. I had decompensation, ups and downs. Since I attended the course, I am having a better control of my glucose. I have learned to adjust my insulin dose regimen, before it seemed difficult to me.”

(23 years old, female, single)

***Continuous glucose monitoring sensor use*:** Eleven patients who wore sensors learned to identify parameters and better amortize the tool. Other patients who had not been interested in using the sensor considered the possibility of starting to use it. Some patients who had few sensor downloads began to increase their percentage of usage and data downloads.

“I didn’t know that the device had so many properties. I learned to program hypoglycemia and hyperglycemia alarms in the course. The nurse taught me how to interpret the trend arrows and how to write down the extra doses of insulin.”

(38 years old, male, single)

“Since the hospital financed me the sensor, I have used it little. Sometimes, it fell off my arm… I didn’t know how to program it… In the course the nurse taught me protectors to prevent the fall and gave me tips to protect it and handle it better. This summer, I will even have it on the beach.”

(24 years old, male, single)

#### 3.2.2. Theme 2: Implementation of Program Recommendations and Difficulties Encountered

During the program, recommendations were offered on the use and storage of medication, in this case, insulin, in addition to reviewing the technique of insulin administration, material preparation, rotation of puncture sites, and replacement of needles.

***Insulin administration*:** Forty-two percent of patients did not rotate the puncture sites correctly to administer insulin. They mostly administered their insulin in their abdomen, and they had a high rate of lipodystrophies. Some patients forgot a dose during the day, or they did not want to administer their insulin in public places.

The administration needle is for single use, and some patients used it frequently, for several doses per day and even for several days.

“I sometimes had nodules in my abdomen. The course nurse taught me that I could avoid it if I alternated the areas where I prick myself. Since the course, I have started to administer insulin in areas of the arms, legs and buttocks. I hope that little by little the nodules will be removed of my abdomen…”

(54 years old, female, married)

“I used the needle of the insulin pen. I was stuck with the same needle for two or three days. The nurse told me that I had to change it after each puncture. I have ordered more needle at the pharmacy and I am trying to remember to change it.”

(48 years old, male, single)

***Food recommendations*:** Fifty-three percent of patients did not have sufficient knowledge to calculate carbohydrate portions of their meals. Some of them did not know the main sources of carbohydrates, fats, and proteins and the benefits of a balanced and healthy diet.

“I admit that I like cakes and sugary products… I don’t like the vegetables… In recent months, I have been trying to do things right and follow the advice that they gave me. It is difficult to change habits, but I am in the process.”

(34 years old, male, single)

“I have never been clear carbohydrate serving counts. Since I went to the course I am learning and trying to adapt me. They also taught me to read the labels on packaged foods.”

(42 years old, female, single)

***Physical activity recommendations*:** Thirty-five percent did not know the performance before performing physical activity. Some of them were afraid of getting lows blood glucose during physical exercise or recognized that they had suffered hypoglycemia doing an intense activity such as playing a soccer game.

“I really like swimming. Sometimes I was afraid to go down in the water, I had a bad time… but I don’t want to stop playing this sport because of diabetes. The nurse advised me to take an extra carbohydrate and adjust the insulin dose before doing the activity.”

(51 years old, female, married)

A 22-year-old single male said, “I once lost consciousness during a soccer game. I didn’t have the glucagon injection. I went to the game without having breakfast; I had a lot of imbalances… In recent games I have done a blood glucose test before playing. I have been using the glucose monitoring sensor for a few months. I am better controlled and I hope I don’t have more scares”.

#### 3.2.3. Theme 3: Influence of Therapeutic Education on Quality of Life and Mood of the Patient

***Quality of life change*:** Sixty-seven percent of participants commented that they had improved their quality of life by attending the education course. They feel more secure and self-sufficient in managing their disease. Something that contributed to a better quality of life.

A 26-year-old single female said, “I have diabetes for more than ten years. Although I have come many times with my mother to the endocrine consultation, I had some problems adjusting my insulin regimen to my diet. I also had difficulties with the use of the glucose monitoring sensor. Thanks to the teaching of the course and the specialist’s team I have managed to clarify some concerns. I feel calmer, more comfortable and surer of myself”.

“A few months ago, I had many hypoglycemias and hyperglycemias. I got up at night with low blood sugar… I had hypoglycemia at work. Since the nurse gave me guidelines to improve my control, I am somewhat better… I am also more confident in what I have to do with my illness.”

(43 years old, male, married)

***Change in mood*:** Eighty-four percent of patients recognized that the chronic disease and the complications complexity generated stress and anxiety. This influenced their usual life, at work, and their interpersonal relationships.

“Since I have better control, I am more animated… I have dared to travel. I have clarified doubts so I am a little more relaxed. I think it is important to understand and treat in this disease because it gives you greater confidence and greatly reduces your anxiety.”

(32 years old, female, single)

“I am eating better; I have learned new things from the sensor and I am trying to do things right. I feel more motivated more eager to go out and willing to take care of myself.”

(41 years old, male, married)

In general, the patients were satisfied with their learning during the course, and they had improved their knowledge and their motivation to implement some changes in their daily life. Patients who experienced an improvement in their glycemic control and self-care reported improvement in their quality of life and mood. Sometimes, diabetic patients suffer anxiety or depressive symptoms associated with their chronic disease, treatment and derived complications.

## 4. Discussion

The authors of this study considered qualitative research as an appropriate design to analyze changes in behavior and learning of diabetic patients after their participation in a protocolized therapeutic education program [23]. In general, the interviewed patients reported words of gratitude to the course and the professionals involved. Most of them finished the course satisfied, and they thought that it had been very useful in improving their self-care at home. As a criticism of the program, several patients commented on the incompatibility of schedules with their respective jobs. As proposals for improvements, some patients commented that they would like to have attended with a family member. The presence of the family member was not included in this study because it can condition the patient’s responses. According to previous evidence, some authors made an educational intervention with the direct relatives of diabetic patients and concluded that it improved their social well-being since they are involved in many cases in managing the disease and also suffer from some problems derived from the disease [15]. The philosophy of our center is that diabetic patients must be autonomous in their disease, but we do plan to conduct future research with different objectives in which family members are included. Above all, this is very important when we work with diabetic adolescents.

Reducing Distress and Enhancing Effective Management for T1D Adults (T1-REDEEM) was a 9-month, randomized control trial for adults with T1D with elevated diabetes distress and HbA1c designed to compare the effectiveness of an intensive education/behavior change intervention with an intervention focused on improving emotion regulation skills. Participants reported how meaningful it was to interact with other adults with T1D. The sense of community that was experienced was significant, even many months after the conclusion of the program; participants recalled that although their worries and fears did not disappear, they were placed in perspective. T1-REDEEM demonstrated that diabetes distress can be successfully addressed among highly distressed adults with T1D with elevated glycemic levels using both educational/behavioral and emotion-focused approaches. It also highlights the importance of emotion regulation, diabetes knowledge, and cognitive skills [24]. In the education program of our study, training was also conducted in small groups; this helps the patients to visualize that they are not alone in this disease and share experiences and concerns. In the results of the qualitative interviews, it was found that the increase in knowledge of diabetes management and the implementation of improvements in their lifestyle had positive effects on their psychosocial well-being.

Other authors tested the hypothesis that nurse-led DSME program is effective in improving lifestyle, clinical, and psychosocial outcomes. Participants in the intervention group showed significant improvements in glycemic control, efficacy, outcome expectation, self-management behaviors, and social support. The compliance and satisfaction with the program were generally high [25]. These data contrast with our study, in which patient satisfaction was high and adherence to a healthy lifestyle focused on diet and physical activity improved.

A study whose objective was to evaluate the effectiveness of a nurse coaching program using motivational interviewing paired with mobile health (*mHealth*) technology on diabetes self-efficacy and self-management demonstrated the short-term effectiveness of this intervention. However, by 9 months, although physical activity remained above the baseline, the improvements in self-efficacy were not sustained. The authors recognized that further research should evaluate the minimum dose of coaching required to continue progress after active intervention and the potential of technology to provide effective ongoing automated reinforcement for behavior change [26]. In our study, the evaluation was made after three months, and adherence to the physical activity recommendations was observed. Perhaps, if evaluation is made after 9 months post-intervention, the patients have lost some habits. It is important to maintain motivation; for this reason, in the Endocrinology Unit of our hospital, educational reinforcement is made every time the patients attend any check-up of their diabetes.

### Limitations

This study has a sample selection bias due to convenience sampling, frequently used in qualitative studies. The patients included are active and participatory people; some subjects who were offered to participate refused due to hesitation, shyness, or difficulties in oral expression.

This study focused on qualitative data. Currently, the authors are conducting a quantitative study in this area in order to triangulate study results. Last, the validity and reliability of qualitative study are represented by trustworthiness, which is achieved by credibility, transferability, and dependability. For future studies, it is intended to make the therapeutic education program protocol and subsequent qualitative and quantitative studies in different hospitals in Spain.

## 5. Conclusions

This qualitative study provides several insights regarding the health education benefits in learning and behavior changes in self-care of patients with type 1 diabetes. Despite years of evolution of diabetes in some patients, a lack of knowledge and self-care has been observed. Therefore, it is vitally important to make therapeutic education programs and, if necessary, make specific sessions of educational reinforcement from time to time. In terms of satisfaction, the majority of patients reported a good perception of the quality of the organization, the professionals involved, and the contents of the program.

## Data Availability

Data are not available out of respect for the protection of patient data. If a reader has any questions or comments, he/she can contact the corresponding author.

## Supplementary Materials

The following supporting information can be downloaded at: https://www.mdpi.com/article/10.3390/healthcare10081375/s1, Supplementary Material File S1: COREQ (COnsolidated criteria for REporting Qualitative research) Checklist.
